# An empirical model for estimating daily atmospheric column-averaged CO_2_ concentration above São Paulo state, Brazil

**DOI:** 10.1186/s13021-022-00209-7

**Published:** 2022-06-11

**Authors:** Luis Miguel da Costa, Gustavo André de Araújo Santos, Alan Rodrigo Panosso, Glauco de Souza Rolim, Newton La Scala

**Affiliations:** 1 Departament of Engineering and Exact Sciences, São Paulo State University, Via de Acesso Prof. Paulo Donato Castellane s/n, Jaboticabal, São Paulo, 14884-900 Brazil; 2 Campus Avançado Porto Franco, Instituto Federal de Educação, Ciência e Tecnologia do Maranhão – IFMA, Rua Custódio Barbosa, no 09, Centro, Porto Franco, Maranhão 65970-000 Brazil; 3 Center of Agricultural, Natural and Literary Sciences, State University of the Tocantina Region of Maranhão (UEMASUL), Av. Brejo do Pinto, S/N – Brejo do Pinto, Estreito, Maranhão 65975-000 Brazil

**Keywords:** Carbon cycle, Remote sensing, OCO-2, Stepwise regression analysis, Climate change, Meteorology

## Abstract

**Background:**

The recent studies of the variations in the atmospheric column-averaged CO_2_ concentration (${\text{X}}_{{{\text{CO}}_{{2}} }}$) above croplands and forests show a negative correlation between ${\text{X}}_{{{\text{CO}}_{{2}} }}$and Sun Induced Chlorophyll Fluorescence (SIF) and confirmed that photosynthesis is the main regulator of the terrestrial uptake for atmospheric CO_2_. The remote sensing techniques in this context are very important to observe this relation, however, there is still a time gap in orbital data, since the observation is not daily. Here we analyzed the effects of several variables related to the photosynthetic capacity of vegetation on ${\text{X}}_{{{\text{CO}}_{{2}} }}$ above São Paulo state during the period from 2015 to 2019 and propose a daily model to estimate the natural changes in atmospheric CO_2_.

**Results:**

The data retrieved from the Orbiting Carbon Observatory-2 (OCO-2), NASA-POWER and Application for Extracting and Exploring Analysis Ready Samples (AppEEARS) show that Global Radiation (Qg), Sun Induced Chlorophyll Fluorescence (SIF) and, Relative Humidity (RH) are the most significant factors for predicting the annual ${\text{X}}_{{{\text{CO}}_{{2}} }}$ cycle. The daily model of ${\text{X}}_{{{\text{CO}}_{{2}} }}$ estimated from Qg and RH predicts daily ${\text{X}}_{{{\text{CO}}_{{2}} }}$ with root mean squared error of 0.47 ppm (the coefficient of determination is equal to 0.44, p < 0.01).

**Conclusion:**

The obtained results imply that a significant part of daily ${\text{X}}_{{{\text{CO}}_{{2}} }}$ variations could be explained by meteorological factors and that further research should be done to quantify the effects of the atmospheric transport and anthropogenic emissions.

**Supplementary Information:**

The online version contains supplementary material available at 10.1186/s13021-022-00209-7.

## Background

Understanding the variability of atmospheric carbon dioxide (CO_2_) concentration in time and space is a crucial task so that we can adopt mitigation strategies. In this sense, several studies analyze the average concentration of this greenhouse gas not only on a global scale [1, 2] but also to estimate anthropogenic emissions in urban centers [3, 4]. Other studies focus on understanding the column-averaged of carbon in the atmosphere (${\text{X}}_{{{\text{CO}}_{{2}} }}$) above tropical forests [5], or above agriculture crops in different seasons of the year [6, 7].

In a recent regional study, da Costa et al. [7] analyze the spatio-temporal variability of ${\text{X}}_{{{\text{CO}}_{{2}} }}$ in a sugarcane-producing area in the southeast region of Brazil. They observed an important inverse relationship between the average carbon concentration in the atmosphere with climatic and vegetative variables. Concluding that the dependence of the natural carbon cycle is related to the predominant agriculture crop in the region and how Global Radiation (Qg), relative humidity (RH), and the Sun Induced Chlorophyll Fluorescence (SIF) was related to this behavior. Similarly, Morais Filho et al. [6] conducted a study that analyzed three different crops and the temporal variability of ${\text{X}}_{{{\text{CO}}_{{2}} }}$ and SIF in these environments, they also found a significant negative correlation between these variables.

However, there is still a temporal gap in the ${\text{X}}_{{{\text{CO}}_{{2}} }}$ data collected by remote sensing, since the measurements are not daily [8, 9]. This type of measurement is important to several factors, such as, estimate the potential capability of atmospheric CO_2_ assimilation by vegetation, establishing public strategies at local levels for climate adaptation and mitigation, and even in economy incorporating daily trends in the carbon market and ecosystems services payments [10–16].

Daily CO_2_ measurements can be made using the Eddy Covariance technique [17–19], although this has the disadvantage of being a point (local) study. In this sense using orbital data, such as Orbiting Carbon Observatory-2 (OCO-2), has become more common [1, 2]. Remote sensing data also can be used to estimate the daily variations of different aspects (e.g., climate, meteorological, land-use changes, ecosystems services) for a larger area [16, 20–23].

Several studies confirm that photosynthesis is the main regulator of atmospheric carbon sinks [10, 24–26]. However, photosynthesis is a process sensitive to climatic variations such as relative humidity [27], precipitation [28], evapotranspiration [29], and incident solar irradiance [30].

Therefore, the natural cycle of CO_2_ is dependent on several aspects, such as vegetation and climate, being necessary data from several different bases for understanding this dynamic [7], turning pre-processing techniques and analysis of autocorrelations necessary, since the multicollinearity introduces an uncertainty due to the model overfit [31, 32]. In this sense, we aim to model the atmospheric CO_2_ cycle above the state of São Paulo to estimate the time changes on a daily scale, based on vegetative and climatic variables retrieved from different orbital platforms, applying a technique to remove the collinearity and after employing a stepwise forward selection, improving in this way the regional understanding of CO_2_.

One of our assumptions, is given that we detrend the ${\text{X}}_{{{\text{CO}}_{{2}} }}$ to maintain only the variability related to the natural interactions [6, 33], the transport by the wind in the atmosphere is not significant, other studies such Hakkarianen et al. [34], that proposed an anomaly model of ${\text{X}}_{{{\text{CO}}_{{2}} }}$, also disregard the atmospheric and wind transport in their study, however, this introduces a limitation of our approach that not account this aspect [35]. In the same way, the trend and increase due to anthropogenic sources are also simplified by this detrend.

## Results

**Fig. 1:**
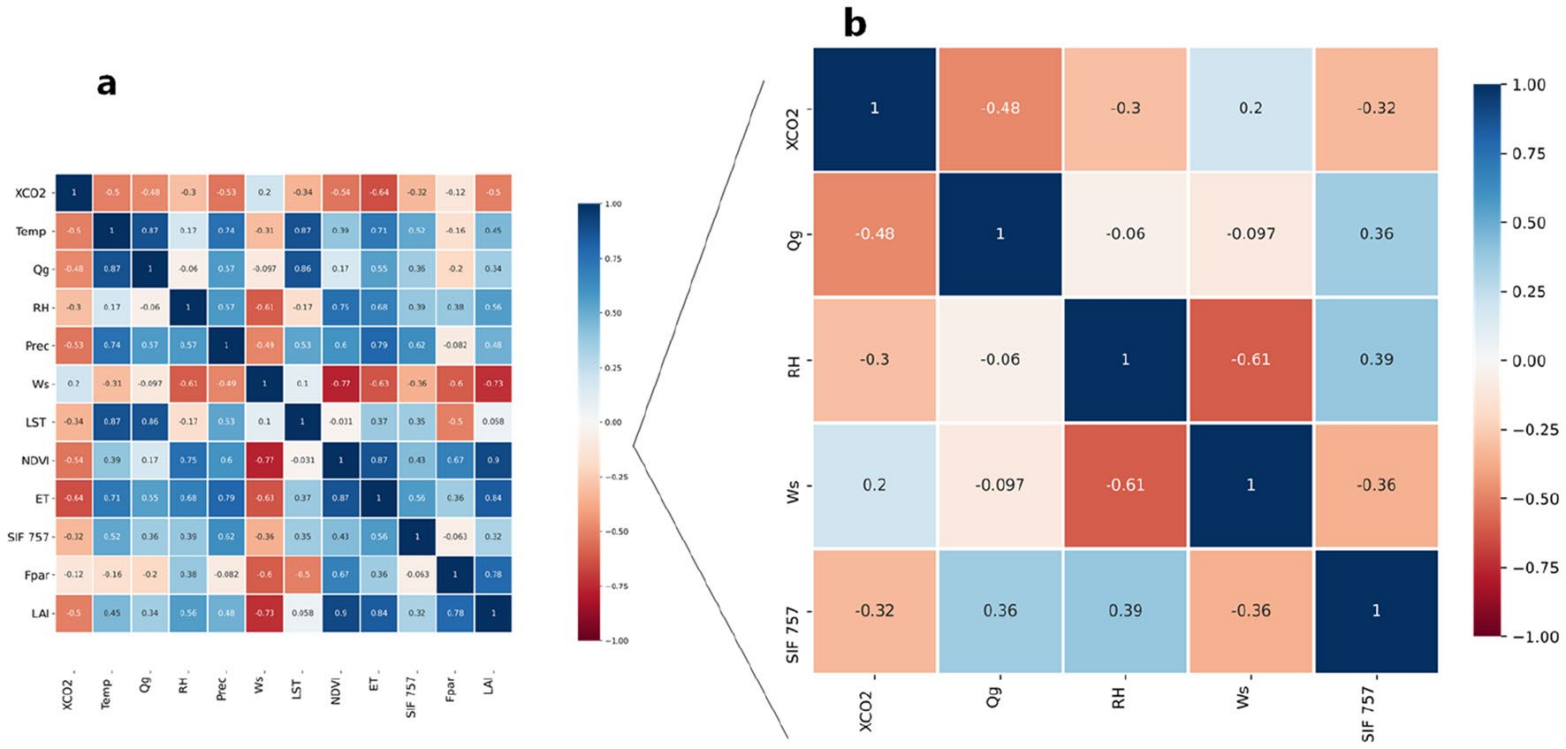
Heatmap of the Pearson’s correlation matrix, where: **a** before the Variance Inflation Factor (VIF) selection and **b** after the selection by Variance Inflation Factor (VIF)

Variance Inflation Factor (VIF) analysis (Table 1) shows it was possible to reduce the number of variables related to ${\text{X}}_{{{\text{CO}}_{{2}} }}$ (according to the adopted criterion, VIF < 10) as shown comparing Fig. 1a with b, before and after the selection, respectively, and therefore reducing the overfit source of uncertainty. Despite wind speed (Ws) hade a VIF < 10, the Pearson’s correlation was not significant (p > 0.05). Variables most related to ${\text{X}}_{{{\text{CO}}_{{2}} }}$ were the Global Radiation (Qg), Sun-Induced chlorophyll Fluorescence at 757 nm (SIF 757), and Relative Humidity (RH).

**Table 1:** Variance Inflation Factor (VIF) of the studied variables

Variable	VIF
**Qg**	**9.35**
**RH**	**5.10**
**SIF 757**	**1.81**
Prec	10.13
Temp	21.43
Ws	4.06
LST	19.54
NDVI	22.15
LAI	87.21
Fpar	65.40
ET	33.47

*Qg* Global radiation, *RH* Relative humidity, *SIF 757* Solar-Induced Chlorophyl Fluorescence at 757 nm, *Prec* Precipitation, *Temp* Temperature at 2 m, *Ws* Wind Speed, *LST* Land Surface Temperature (MODIS), *NDVI* Normalized Difference Vegetation Index, *LAI* Leaf Area Index, *ET* Evapotranspiration

**Fig. 2:**
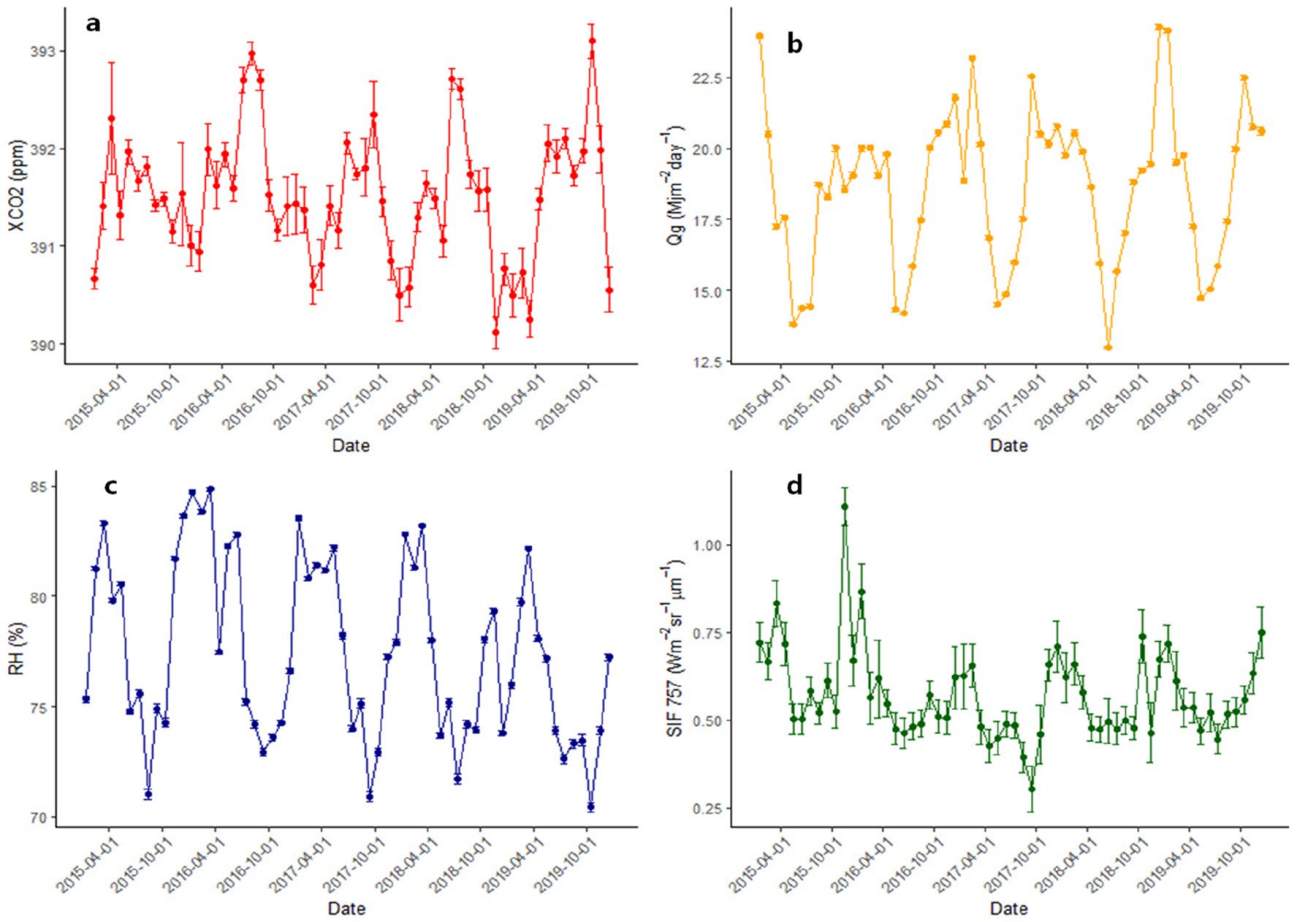
Monthly variability of ${\text{X}}_{{{\text{CO}}_{{2}} }}$ (**a**), Qg (**b**), RH (**c**), and SIF 757 (**d**) over the period from January 2015 to December 2019. Where ${\text{X}}_{{{\text{CO}}_{{2}} }}$ column average of carbon dioxide in the atmosphere (ppm), *Qg* global radiation (MJ m^−2^ day^−1^), *RH* relative humidity (%), *SIF 757* sun-induced chlorophyll fluorescence at 757 nm (Wm^−2^ sr^−1^ μm^−1^)

Regarding the temporal variability of ${\text{X}}_{{{\text{CO}}_{{2}} }}$, the maximum mean for the analyzed period was 393.09 ± 0.17 ppm and occurred in October 2019, while the minimum average was in November 2018, being 390.11 ± 0.15 ppm (Fig. 2a). Meanwhile, the Qg (Fig. 2b) ranged between 24.3 ± 0.09 and 13.07 ± 0.04 (MJ m^−2^ day^−1^), with the maximum average occurring in December 2018 and the minimum in June of the same year.

SIF 757 (Fig. 2d) had the highest average recorded in the period in November 2015 [1.1 ± 0.05 (Wm^−2^ sr^−1^ μm^−1^)] and the lowest in September 2017 [0.3 ± 0.06 (Wm^−2^ sr^−1^ μm^−1^)], while the Relative Humidity (Fig. 2c) ranged from 84.86 ± 0.07 to 70.44 ± 0.19%, where the highest mean was observed in March 2016 and the lowest in October 2019.

Regarding SIF 757 the minimum averages occurred in June of 2015 and 2016, September 2017, November 2018, and July 2019, ranging from 0.3 to 0.46 Wm^−2^ sr^−1^ μm^−1^ (Fig. 2d). The minimum Qg averages vary between May and June for the entire series approximately between 13.07 and 14.71 MJ m^−2^ day^−1^ (Fig. 2b). Maximum Qg averages are concentrated between December and January of each year, reaching 24 MJ m^−2^ day^−1^ in those months (Fig. 2b). The maximum average of SIF 757 occurs between November and February of each year, ranging from 0.8 to 1.1 Wm^−2^ sr^−1^ μm^−1^ (Fig. 2d).

**Fig. 3:**
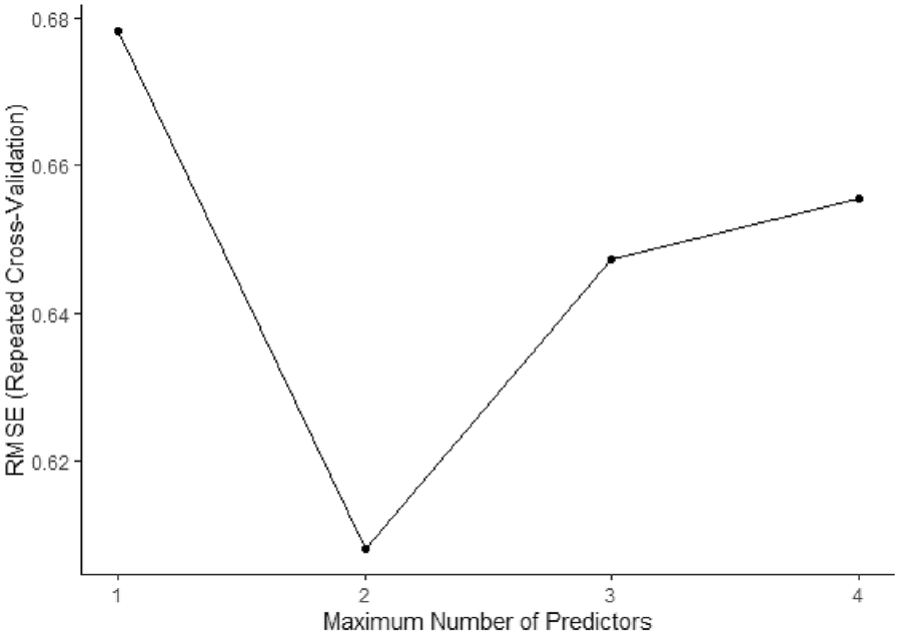
RMSE score for the training sample

The stepwise forward selection method, with multiple cross-validation, had the best result with two variables, with a root mean squared error (RMSE) of ~ 0.60 ppm in the training sample (Fig. 3), the selected variables being Qg and RH, respectively (Eq. 1).

1 $$X_{{CO}_{2\ (daily)}} = 391.484\,\left( { \pm \,0.89} \right) - \left( { \pm \,0.089} \right) \times Qg - 0.263 \left( { \pm \,0.09} \right) \times RH$$

**Fig. 4:**
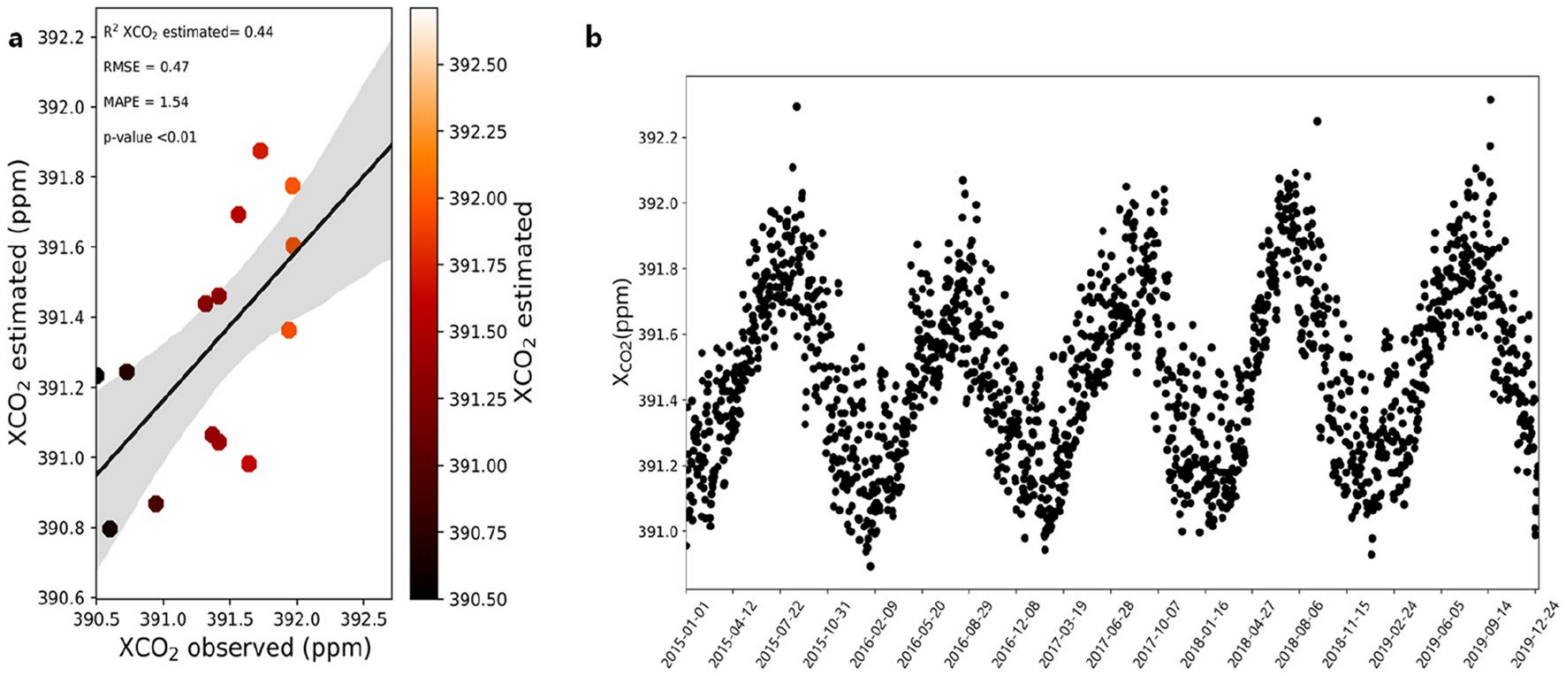
**a** Cross-validation between ${\text{X}}_{{{\text{CO}}_{{2}} }}$ estimated by stepwise and ${\text{X}}_{{{\text{CO}}_{{2}} }}$ observed by OCO-2 and **b** Daily downscale of natural ${\text{X}}_{{{\text{CO}}_{{2}} }}$ using Eq. 1 and daily measurements of NASA/POWER from January 2015 to December 2019

The model built in the training (Eq. 1) was applied in the test sample of the variables cited (Qg and RH), and from the cross-validation of the estimated data with the observed data, we observe an R^2^ of 0.44, the values of the metrics MSE, RMSE, and MAE were 0.22, 0.47, and 0.37 (ppm) respectively, and for MAPE we found a value of 1.54% (p < 0.01) (Fig. 4a), with this we were able to reduce the time scale of the OCO-2 satellite from every 15 days to a daily scale (Fig. 4b).

## Discussion

The natural annual cycle of ${\text{X}}_{{{\text{CO}}_{{2}} }}$ is affected by factors related to climate and vegetation aspects [6, 36, 37]. Due to the VIF analysis, we were able to summarize three main factors for São Paulo state: Global Radiation (Qg), Relative Humidity (RH) and Sun-Induced chlorophyll Fluorescence at 757 nm (SIF 757), reducing the uncertainties in the model formulation since we removed the overfit caused by multicollinearity [31, 32]. Several studies have already been conducted using this method to identify which variables select for ecological studies [38], computational studies [39], and remote sensing studies [40].

Except for wind speed (Ws), all variables studied correlated negatively with ${\text{X}}_{{{\text{CO}}_{{2}} }}$ (Fig. 1), hence, related to the sink of atmospheric CO_2_. The non-significant correlation between ${\text{X}}_{{{\text{CO}}_{{2}} }}$ and Ws could be related to the detrending of the atmospheric CO_2_ concentration (see Methods section), which removes the transport effect and simplify the ${\text{X}}_{{{\text{CO}}_{{2}} }}$ variability only for the biochemical cycle [6, 7, 33]. In general, the highest concentrations of ${\text{X}}_{{{\text{CO}}_{{2}} }}$ are observed in the months corresponding to the Brazilian autumn and winter (April to August) and lowest in the summer, from December to February. Studies such as by Siabi et al. [41] and Falahatkar et al. [42] reported how the different seasons affect the average CO_2_ concentration in the atmosphere.

Recently, researches were conducted at regional scales in Brazil such as by Morais Filho et al. [6] and da Costa et al. [7], indicating negative correlations between ${\text{X}}_{{{\text{CO}}_{{2}} }}$ and SIF over agricultural areas, approximately − 0.5 and − 0.8, respectively. SIF is a variable directly related to the photosynthesis of plants, laboratory-scale experiments have demonstrated this relation [43], and remote sensing studies at the canopy and global level reported positive relations between SIF and Gross Primary Production, and also a negative correlation between SIF and the ${\text{X}}_{{{\text{CO}}_{{2}} }}$ [5, 44–46].

As a result of photosynthesis, it is expected that SIF increases during summer [7, 41], as in this season, higher precipitation events and higher temperatures are observed [47]. Our results show higher SIF average values in the months when summer occurs in the São Paulo state, and an inverse relationship between SIF and ${\text{X}}_{{{\text{CO}}_{{2}} }}$. The lowest average values of ${\text{X}}_{{{\text{CO}}_{{2}} }}$ usually occur during the summer period in the study region. This is due to plant CO_2_ assimilation [48], printing quasi-periodical ${\text{X}}_{{{\text{CO}}_{{2}} }}$_,_ and SIF time changes as well as observed in other studies [5, 6, 41, 49].

Most of São Paulo’s state has a wet summer and dry winter [47] resulting in a positive correlation between precipitation and SIF (Pearson’s correlation = 0.61 and p < 0.05), while negative with ${\text{X}}_{{{\text{CO}}_{{2}} }}$ (r = − 0.49, p < 0.05) (Fig. 1a). Precipitation is a photosynthetic control factor, so the greater availability of water that exists in the summer in São Paulo’s state induces plants to perform more photosynthesis through primary productivity, which leads to a reduction of atmospheric CO_2_. The opposite is observed in the dry winter because water availability is lower resulting in less photosynthesis, or less CO_2_ assimilation by plants, either in natural or agricultural areas [7, 28, 50].

Another effect observed during summer in the region is the increase of relative humidity (RH), which reduces the water transfer between soil or plant to the atmosphere [51], inducing plants to keep their stomata open, where CO_2_ assimilation occurs [52]. Studies have already shown the relationship of stomata opening in periods with good water availability is related to plant growth [53, 54]. Thus, establishing the negative relationship between RH and ${\text{X}}_{{{\text{CO}}_{{2}} }}$, also previously observed by Golkar et al. [27].

In the same way, another requirement for photosynthesis occurs is sunlight, which is the source of energy to carry out the biochemical processes of this phenomenon. Therefore, as the amount of radiation (Qg) is absorbed by the plant, photosynthesis tends to increase, and consequently higher CO_2_ assimilation, decreasing in this way the concentration of this greenhouse gas in the atmosphere [7, 30]. We can observe these relationships in our results (Fig. 3b), Qg correlates positively with SIF, and these variables relate negatively with ${\text{X}}_{{{\text{CO}}_{{2}} }}$.

Since we are dealing with the natural annual cycle of CO_2_ the main factor of the higher concentrations of this gas in the atmosphere is due to the lowest photosynthetic absorption by plants. The autumn and winter have low available water and sunlight for plants, leading to a decrease in photosynthesis, also another important factor is that the annual calendar for agriculture in the state of São Paulo has harvest periods between these seasons [55], and as consequence decreasing the cover area by vegetation. Shekhar et al. [56] show how the crop’s grown in summer decrease the values of ${\text{X}}_{{{\text{CO}}_{{2}} }}$ over the Nile Delta and when the harvest starts the values of ${\text{X}}_{{{\text{CO}}_{{2}} }}$ are higher, also, they found that SIF values are higher in the grown season.

Our model was based on Qg and RH, which are two variables related to the CO_2_ assimilation process, or CO_2_ sink. The model has lower RMSE values than have been reported in previous studies, such as by Guo et al. [57] where the values of this metric ranged from 0.7 to 1.1 ppm. In a more recent study by Taylor et al. [58] when evaluating initial OCO-3 data results from the globe and model-related errors, they found an RMSE between 1 and 2 ppm. Another important measure is the MAPE, which shows in percentage how much we are getting wrong, studies with remote sensing have already demonstrated errors below 10% as being considered extremely low for predicting plant and climate aspects [59, 60]. With this, we can evaluate that the performance of the model proposed in this work presents a very low error.

**Fig. 5:**
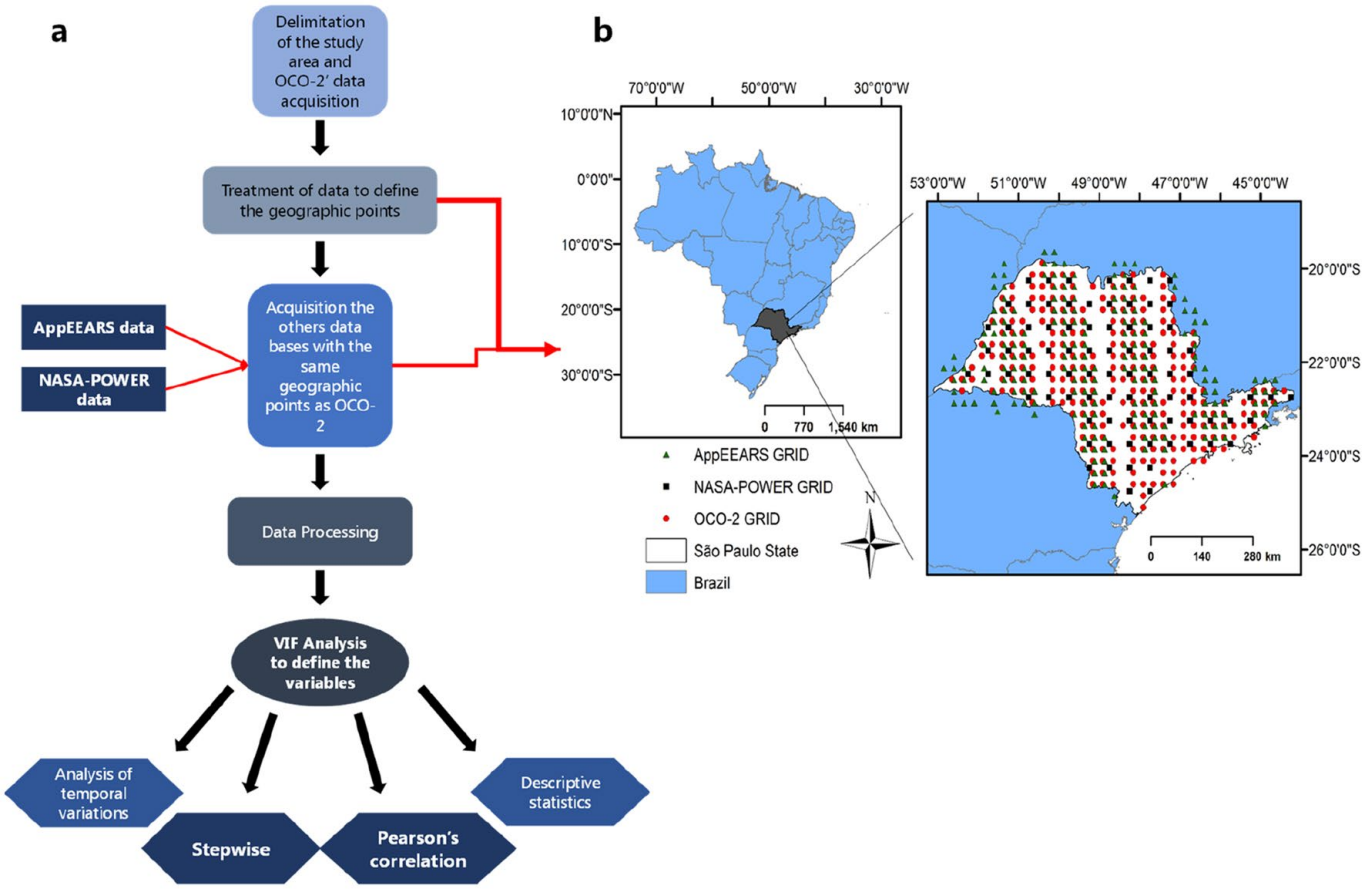
Flowchart of data acquisition, processing, analysis (**a**), and sounding map of the satellite observations in the study region (**b**). Where red dots represent the OCO-2 soundings, the black dots represent the NASA-POWER and the dark green represent the MODIS

The coefficient of determination (R^2^) was 0.44, an increment of almost 20% from the simple linear fit with Qg alone, which has a higher importance in the model. Although the R^2^ is moderate, studies using other orbital sensors such as MODIS to model the average CO_2_ concentration in the atmosphere have reported similar results [23]. In addition, we should consider that although OCO-2 and NASA-POWER are two high quality and validated databases [8, 9, 61], the difference between grids and spatial resolution (see Table 2 in Methods and Fig. 5b) cannot be disregarded, as it is an aspect that can influence these results, leading us to consider the coefficient of determination observed in this study as being high.

These differences between the databases can be suppressed by the greater temporal coverage of NASA-POWER, allowing us to estimate the daily temporal variability of the natural CO_2_ cycle in the atmosphere for the state of São Paulo, besides reducing in the future the spatial scale of ${\text{X}}_{{{\text{CO}}_{{2}} }}$ obtained from OCO-2 and gaining greater spatial resolution cover. Other vegetation index-based models aimed at reducing the spatial sampling of OCO-2 data, but focused on SIF, as is the case of Zhang et al. [62] and Yu et al. [63].

Despite the errors associated with the model and the uncertainty measures due to the difference in satellite resolution, an advantage of using models similar to the one proposed here is being able to have a daily measure of the variability of atmospheric CO_2_ and how the climate parameters affect this dynamic, also serving as an indirect indicator of how is the daily assimilation capacity of this gas in a region.

## Conclusions

In summary, the cycle of ${\text{X}}_{{{\text{CO}}_{{2}} }}$ in the state of São Paulo has higher average values during April to October, periods of lower intensity of rainfall, and is considered as the winter in the state, in the other hand the lowest averages of ${\text{X}}_{{{\text{CO}}_{{2}} }}$ were usually observed between December to March, this period corresponds to the summer, and the inverse behavior was observed for SIF 757, global radiation (Qg) and relative humidity (RH). This pattern is due to the relationship between photosynthesis and Carbon assimilation, given that photosynthesis is a process sensitive to climate variation and a process that depends on water and light, in summer this process tends to be greater, leading to a decrease in CO_2_.

Concerning the daily ${\text{X}}_{{{\text{CO}}_{{2}} }}$ model presented, it performed well when we looked at the set of metrics presented. Given this, we were able to estimate the daily behavior of natural ${\text{X}}_{{{\text{CO}}_{{2}} }}$ in general for the state of São Paulo, a semi-periodical wave with a maximum peak between March and July, and a minimum peak between December to February. There are still challenges in this aspect, such as the transport process in the atmosphere, which was simplified due to the detrend in the dataset, that also remove the anthropogenic sources in the CO_2_ cycle, however, this study was capable in advance in the temporal gap, and properly address how to estimate the natural behavior of this gas in a synthetic way using daily meteorological open access data, establishing a low-cost approach, and we believe that this study will serve as a basis for further implementations.

We suggest that for future work, the relationship between soil respiration and factor controlling organic matter decay in soil with the ${\text{X}}_{{{\text{CO}}_{{2}} }}$ would be needed to better understand CO_2_ dynamics, as well the addition of variables related to activities, such as in transports or the data of fossil fuel consumption, in big cities to improve predictions, as well the atmospheric transport.

## Methods

### Study region

The state of São Paulo (SP) (Fig. 5b), southern Brazil, has approximately 249 × 10^3^ km^2^ and 645 municipalities, with a demographic density of 179.84 habitants/km^2^ [64] being one of the main agricultural hubs of Brazil, regarding the production of sugarcane and citrus [65]. According to Rolim et al. [47] the climate of the state, in general, has its areas characterized by a humid subtropical climate with dry winter, followed by humid tropical dry winter and sub-humid tropical dry winter, according to the climate classification proposed by Camargo [66].

### Products of remote sensing: acquisition and processing

Greenhouse gas, climate, and vegetation data were collected from different satellites (Table 2) for a time series from 2015 to 2019 and were aggregated on a monthly scale. The primary product of the Orbiting Carbon Observatory-2 (OCO-2) consists of georeferenced estimates of the mean atmospheric CO_2_ concentration (${\text{X}}_{{{\text{CO}}_{{2}} }}$), in addition, the Sun Induced Chlorophyll Fluorescence (SIF), retrieved due to the overlap that occurs in the SIF wavelengths with the O_2_ absorption wavelength (680–850 nm) [8, 9, 43]. Data from this satellite have already been validated by Crisp et al. [8] and, according to O'Dell et al. [9], this satellite provides about 65,000 quality observations per day worldwide.

Here we used the version 9 of the OCO-2 with a bias-correction and considered only the measurements with the best quality flag (quality flag = 0, meaning that has no cloud cover) [67, 68], also, we do not consider the data with more than 12 alert level at nadir viewing [33, 69]. Concerning the SIF, we take into account only the SIF at 757 nm, this was due to previous studies that exploited the relationship in the São Paulo’s State [6, 7] and, also because this wavelength is closer to the far-red peak (~ 740 nm) in the whole SIF signal [43].

MODIS sensor data were extracted from the “Application for Extracting and Exploring Analysis Ready Samples” (AppEEARS). This application allows users to obtain subsets of large databases using spatial and temporal parameters. Two types of sample requests are available: point samples by entering geographic coordinates and area samples using vector polygons. Sample requests submitted to AppEEARS provide users with not only data values but also associated quality data values. Interactive visualizations with summary statistics are provided for each sample within the application, which allows users to view and interact with their samples before downloading the data [70].

Nasa Power data (https://power.larc.nasa.gov ) consists of precipitation (mm), surface solar shortwave irradiance (MJ m−2 day−1), average air temperature (ºC), and relative humidity at 2 m (%). This platform consists of a NASA project entitled: Worldwide Energy Resource Forecast (POWER) and was initiated to enhance the current renewable energy dataset and create new datasets from new satellite systems﻿ [71].

**Table 2:** Studied variables, data base, temporal and spatial resolution

Variable	Data base	Temporal resolution	Spatial resolution
GHG			
${\text{X}}_{{{\text{CO}}_{{2}} }}$ (ppm)	OCO-2 “OCO-2 Data product user’s guide, 2016” V9	16 days	1.29 km × 2.25 km
Climate			
Surface solar shortwave irradiance (Global radiation, Qg) (MJ m^−2^ day^−1^)	FLASH Flux Version 3 (A, B,C) NASA/POWER	Daily	111.3 km × 111.3 km
Average air temperature at 2 m (Temp) (ºC)	GEO-5 FP-IT (NASA/POWER)	Daily	111.3 km × 111.3 km
Land surface temperature (LST) (ºC)	MOD11A1.006 V6 MODIS-TERRA	Daily	1200 km × 1200 km
Wind speed at 10 m (WS) (m s^−1^) (ºC)	GEO-5 FP-IT (NASA/POWER)	Daily	111.3 km × 111.3 km
Relative humidity (RH) (%)	GEO-5 FP-IT (NASA/POWER)	Daily	111.3 km × 111.3 km
Precipitation (Prec) (mm day^−1^)	GEO-5 FP-IT (NASA/POWER)	Daily	111.3 km × 111.3 km
Vegetation			
SIF 757	OCO-2 “OCO-2 Data product user’s guide, 2016” V9	16 days	1.29 km × 2.25 km
LAI (m^2^ m^−2^)	MCD15A2H.006 V6 MODIS- CFPAR	8 days	500 m × 500 m
Fraction of Photosynthetically Active Radiation (Fpar) (%)			
Evapotranspiration (ET) (kg m^−2^ day^−1^)	MOD16A2.006 V6 MODIS-TERRA	8 days	500 m × 500 m
NDVI	MOD13A1.006 V6 MODIS-TERRA	16 days	500 m × 500 m

To minimize the differences between the spatial and temporal resolutions of the different orbital sensors used in this study, the process described in Fig. 5a was employed, which establishes a standard for the acquisition of data from the coordinates obtained in the OCO-2 platform (Fig. 5b). We emphasize that several studies have been conducted using different time and spatial scales [6, 7, 27].

### Pre-process of the data

Using the regression method proposed by Gujarati and Potter [72], we removed the trend from ${\text{X}}_{{{\text{CO}}_{{2}} }}$ data, in order to understand the natural and regional variability of ${\text{X}}_{{{\text{CO}}_{{2}} }}$ and its relationships with other factors [6, 7, 33]. The other variables were standardized using the function *scale* from the R language [73].

### Variance Inflation Factor (VIF)

Variance Inflation Factor (VIF) analysis was performed. This analysis is a method of detecting multicollinearity within a database since the relation between the predictors for a multi-regression model can affect the estimative and the standard errors associated with the regression model [31]. The VIF is based on the R^2^ value (Eq. 2), and should not be greater than 10, however, this can vary according to the study [31, 32].

2 $$VIF = \frac{1}{{1 - R^{2} }}$$

where R^2^ is the coefficient of determination.

### Temporal variability, Pearson’s correlation, and dependency analysis

The data was processed using month averages for the analysis period, except precipitation, which consists of monthly sums for the entire state of SP (ST.1). The means were subjected to analysis of variance (F-test) to obtain the mean standard errors. Simultaneously, the basic assumptions of analysis of variance and, normality of errors, and homogeneity of variances were tested for the selected variables by VIF analysis. To understand the variation of ${\text{X}}_{{{\text{CO}}_{{2}} }}$ with the other variables, Pearson correlation analyses were performed. More about the descriptive statics of selected variables in VIF, such as the number of observations (soundings) for each month, can be found in Additional file 1: Table S2

### Stepwise: forward selection

**Fig. 6:**
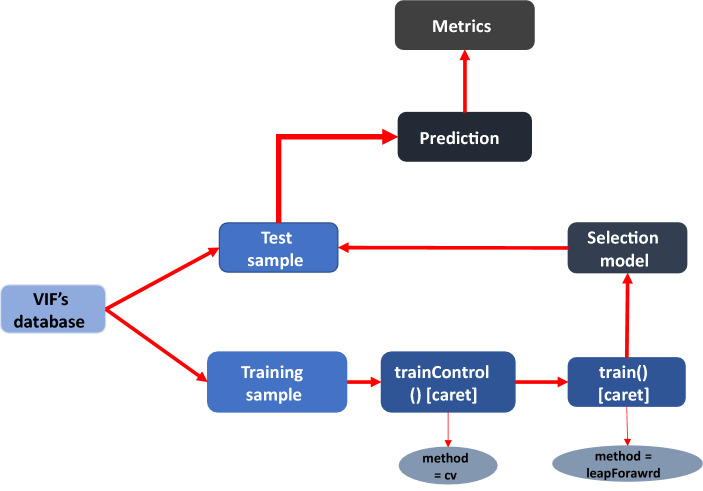
Flowchart of the stepwise construction

The stepwise method used in this study was the forward selection method being performed in R language [73], as can be seen in the flow chart (Fig. 6), the variables selected in the VIF analysis were separated into a training and test samples (70% and 30% of the dataset respectively). The training sample was submitted to the *train ()* function of the *caret* package, using repeated cross-validation (cv) method. This technique consists in randomly splitting the training dataset into k-subsets, one of them is reserved and the model is trained with the others, and after is validated with the reserved subset, this process is repeated until each subset serves as a test sample, finally, the average error is how the performance is given [74]. The model is based on the lowest Root Mean Squared Error (RMSE) and, from variables selected in training, the generated model is applied to the test sample defined at begging for estimating the ${\text{X}}_{{{\text{CO}}_{{2}} }}$ with these independent data. Finally, cross-validation between the estimated data and observed data in the test sample was performed and from this, we derive the metrics Mean squared error (MSE), Root Mean Squared Error (RMSE), Mean Absolute Error (MAE), R^2^, and Mean absolute percentage error (MAPE).

## Supplementary Information

**Additional file 1: Table S1.** Variables used in the VIF analyses. These variables were standarlized by scale() in R language. **Table S2. ** Descreptive statistics.

## Data Availability

The processed data can be found in Additional file tables attached to this paper. The ${\text{X}}_{{{\text{CO}}_{{2}} }}$ and SIF were retrieved from: https://co2.jpl.nasa.gov/build?mission=oco-2&dataset=OCO2L2Stdv10; The MODIS products from: https://lpdaacsvc.cr.usgs.gov/appeears/; the NASA-POWER from: https://power.larc.nasa.gov/; The VIF and regression was made in R language and the code is available at: https://github.com/lm-costa/stepwise_fapesp.

## Abbreviations

AppEEARS: Application for extracting and exploring analysis ready samples
CO_2_: Carbon dioxide
ET: Evapotranspiration
LAI: Leaf area index
LST: Land surface temperature (MODIS)
MAE: Mean absolute error
MAPE: Mean absolute percentage error
MSE: Mean squared error
NASA-POWER: NASA project entitled: worldwide energy resource forecast
NDVI: Normalized difference vegetation index
OCO-2: Orbiting carbon observatory-2
OCO-3: Orbiting carbon observatory-3
Prec: Precipitation
Qg: Global radiation
RH: Relative humidity
RMSE: Root mean squared error
SIF 757: Solar-induced chlorophyl fluorescence at 757 nm
Temp: Temperature at 2 m
VIF: Variance Inflation Factor
Ws: Wind speed at 2 m
${\text{X}}_{{{\text{CO}}_{{2}} }}$: Column average of carbon dioxide in the atmosphere

## References

[1] Eldering A, O'Dell CW, Wennberg PO, Crisp D, Gunson MR, Viatte C, Avis C, Braverman A, Castano R, Chang A, Chapsky L, Cheng C, Connor B, Dang L, Doran G, Fisher B, Frankenberg C, Fu D, Granat R, Hobbs J, Lee RAM, Mandrake L, McDuffie J, Miller CE, Myers V, Natraj V, O'Brien D, Osterman GB, Oyafuso F, Payne VH, Pollock HR, Polonsky I, Roehl CM, Rosenberg R, Schwandner F, Smyth M, Tang V, Taylor TE, To C, Wunch D, Yoshimizu J (2017). The Orbiting Carbon Observatory-2: first 18 months of science data products. Atmos. Meas. Tech..

[2] Boesch H, Baker D, Connor B, Crisp D, Miller C (2011). Global characterization of CO_2_ column retrievals from shortwave-infrared satellite observations of the orbiting carbon observatory-2 mission. Remote Sensing.

[3] Ye X, Lauvaux T, Kort EA, Oda T, Feng S, Lin JC, Yang EG, Wu D (2020). Constraining fossil fuel CO_2_ emissions from urban area using OCO-2 observations of total column CO_2_. J Geophys Res Atmos.

[4] Kort EA, Frankenberg C, Miller CE, Oda T (2012). Space-based observations of megacity carbon dioxide. Geophys Res Lett.

[5] Parazoo NC, Bowman K, Frankenberg C, Lee JE, Fisher JB, Worden J, Jones DBA, Berry J, Collatz GJ, Baker IT, Jung M, Liu J, Osterman G, O’Dell C, Sparks A, Butz A, Guerlet S, Yoshida Y, Chen H, Gerbig C (2013). Interpreting seasonal changes in the carbon balance of southern Amazonia using measurements of $${\text{X}}_{{{\text{CO}}_{{2}} }}$$ and chlorophyll fluorescence from GOSAT. Geophys Res Lett.

[6] Morais Filho LFF, de Meneses KC, de Santos GA, da Bicalho AE, de Rolim GS, La Scala N (2021). $${\text{X}}_{{{\text{CO}}_{{2}} }}$$ temporal variability above Brazilian agroecosystems: a remote sensing approach. J Environ Manage.

[7] da Costa LM, de Araújo Santos GA, de Mendonça GC (2021). Spatiotemporal variability of atmospheric CO_2_ concentration and controlling factors over sugarcane cultivation areas in southern Brazil. Environ Dev Sustain.

[8] Crisp D, Fisher BM, O’dell C, Frankenberg C, Basilio R, Bösch H, Brown LR, Connor B, Deutscher NM, Eldering A, Griffith D, Gunson M, Kuze A, Mandrake L, Mcduffie J, Messerschmidt J, Miller CE, Morino I, Natraj V, Notholt J, O’Brien DM, Oyafuso F, Polonsky I, Robinson J, Salawitch R, Sherlock V, Smyth M, Suto H, Taylor TE, Thompson DR, Wennberg PO, Wunch D, Yung YL (2012). The ACOS CO_2_ retrieval algorithm—Part II: global $${\text{X}}_{{{\text{CO}}_{{2}} }}$$ data characterization. Atmos Meas Tech.

[9] O’Dell CW, Connor B, Bösch H, O’Brien D, Frankenberg C, Castano R, Christi M, Eldering D, Fisher B, Gunson M, McDuffie J, Miller CE, Natraj V, Oyafuso F, Polonsky I, Smyth M, Taylor T, Toon GC, Wennberg PO, Wunch  D (2012). Description and validation against synthetic observations. The ACOS CO_2_ retrieval algorithm—Part 1. Atmos Meas Tech.

[10] Li X, Xiao J, Fisher JB, Baldocchi DD (2021). ECOSTRESS estimates gross primary production with fine spatial resolution for different times of day from the International Space Station. Remote Sensing Environ.

[11] Wassie YT, Adaramola MS (2021). Socio-economic and environmental impacts of rural electrification with solar photovoltaic systems: evidence from southern Ethiopia. Energy Sustainable Develop.

[12] Rupp M, Rieke C, Handschuh N, Kuperjans I (2020). Economic and ecological optimization of electric bus charging considering variable electricity prices and CO_2_ eq intensities. Transp Rese D Transp Environ.

[13] Smith P, Calvin K, Nkem J (2020). Which practices co-deliver food security, climate change mitigation and adaptation, and combat land degradation and desertification?. Glob Change Biol.

[14] Li Q, Wu S, Lei Y, Li S (2020). Dynamic features and driving forces of indirect CO_2_ emissions from Chinese household: a comparative and mitigation strategies analysis. Sci Total Environ.

[15] Cernusak LA (2020). Gas exchange and water-use efficiency in plant canopies. Plant Biol.

[16] Parras R, de Mendonça GC, Araújo Costa RC, Pissarra TCT, Valera CA, Fernandes LFS, Leal Pacheco FA (2020). The configuration of forest cover in Ribeirão Preto: a diagnosis of Brazil’s forest code implementation. Sustainability.

[17] de Oliveira ML, dos Santos CAC, de Oliveira G, Perez-Marin AM, Santos CAG (2021). Effects of human-induced land degradation on water and carbon fluxes in two different Brazilian dryland soil covers. Sci Total Environ.

[18] Cabral OMR, Freitas HC, Cuadra SV, de Andrade CA, Ramos NP, Grutzmacher P, Galdos M, Packer APC, da Rocha HR, Rossi P (2020). The sustainability of a sugarcane plantation in Brazil assessed by the eddy covariance fluxes of greenhouse gases. Agric For Meteorol.

[19] Oliveira RR, Pezzi LP, Souza RB, Santini MF, Cunha LC, Pacheco FS (2019). First measurements of the ocean-atmosphere CO_2_ fluxes at the Cabo Frio upwelling system region, Southwestern Atlantic Ocean. Cont Shelf Res.

[20] Tedesco D, de Oliveira MF, dos Santos AF, Costa Silva EH, de Souza Rolim G, da Silva RP (2021). Use of remote sensing to characterize the phenological development and to predict sweet potato yield in two growing seasons. Eur J Agron.

[21] de Aparecido LE, Lorençone PA, de Rolim GS, de Meneses KC, da de Moraes JRSC, Torsoni GB (2021). Can nonlinear agrometeorological models estimate coffee foliation?. J Sci Food Agric.

[22] Helman D, Lensky IM, Osem Y, Rohatyn S, Rotenberg E, Yakir D (2017). A biophysical approach using water deficit factor for daily estimations of evapotranspiration and CO_2_ uptake in Mediterranean environments. Biogeosciences.

[23] Guo M, Xu J, Wang X, He H, Li J, Wu L (2015). Estimating CO_2_ concentration during the growing season from MODIS and GOSAT in East Asia. Int J Remote Sens.

[24] Li X, Hu X-M, Cai C, Jia Q, Zhang Y, Liu J (2020). Terrestrial CO_2_ fluxes, concentrations, sources and budget in Northeast China: observational and modeling studies. J Geophysic Res Atmos.

[25] Chhabra A, Gohel A (2019). Dynamics of atmospheric carbon dioxide over different land cover types in India. Environ Monit Assess.

[26] Walker AP (2015). Predicting long-term carbon sequestration in response to CO_2_ enrichment: how and why do current ecosystem models differ?. Global Biogeochem Cycles.

[27] Golkar F (2020). Using OCO-2 satellite data for investigating the variability of atmospheric CO_2_ concentration in relationship with precipitation, relative humidity, and vegetation over Oman. Water.

[28] Barbosa HA, Lakshmi KumarSilva TVLRM (2015). Recent trends in vegetation dynamics in the South America and their relationship to rainfall. Nat Hazards.

[29] Wagle P, Gowda PH, Billesbach DP, Northup BK, Torn MS, Neel JPS, Biraud SC (2020). Dynamics of CO and HO fluxes in Johnson grass in the US Southern Great Plains. Sci Total Environ.

[30] Liu Q, Fu YH, Zeng Z, Huang M, Li X, Piao S (2016). Temperature, precipitation, and insolation effects on autumn vegetation phenology in temperate China. Global Change Biol.

[31] Miles J. Tolerance and variance inflation factor. Wiley StatsRef: Statistics Reference Online. 2014.10.1002/9781118445112.STAT06593.

[32] Kalnins A (2018). Multicollinearity: how common factors cause Type 1 errors in multivariate regression. Strategic Manage J.

[33] Rossi FS, de Araújo Santos GA, de Souza Maria L, Lourençoni T, Pelissari TD, Della-Silva JL, Júnior JWO, de Silva  AA, Lima M, Teodoro PE, Teodoro LPR, de Oliveira-Júnior JF, LaScala N, da Junior SCA (2022). Carbon dioxide spatial variability and dynamics for contrasting land uses in central Brazil agricultural frontier from remote sensing data. J South Am Earth Sci.

[34] Hakkarainen J, Ialongo I, Tamminen J (2016). Direct space-based observations of anthropogenic CO_2_ emission areas from OCO-2. Geophysic Res Lett.

[35] Wu D, Lin J, Fasoli B, Oda T, Ye X, Lauvaux T, Yang E, Kort E (2018). A Lagrangian approach towards extracting signals of urban CO_2_ emissions from satellite observations of atmospheric column CO_2_ ($${\text{X}}_{{{\text{CO}}_{{2}} }}$$): X-stochastic time-inverted Lagrangian transport model (“X-STILT v1”). Geosci Model Develop.

[36] Sá MMF, Schaefer CEGR, Loureiro DC, Simas FNB, Alves BJR, de Sá Mendonça E, de Figueiredo EB, La Scala N, Panosso AR (2019). Fluxes of CO_2_, CH_4_, and N_2_O in tundra-covered and Nothofagus forest soils in the Argentinian Patagonia. Sci Total Environ.

[37] Vicentini ME, Pinotti CR, Hirai WY, de Moraes MLT, Montanari R, Filho MCMT, Milori DMBP, Júnior NLS, Panosso AR (2019). CO_2_ emission and its relation to soil temperature, moisture, and O_2_ absorption in the reforested areas of Cerrado biome Central Brazil. Plant Soil.

[38] Graham MH (2003). Confronting multicollinearity in ecological multiple regression. Ecology.

[39] Tamura R, Kobayashi K, Takano Y, Miyashiro R, Nakata K, Matsui  T (2019). Mixed integer quadratic optimization formulations for eliminating multicollinearity based on variance inflation factor. J Global Optim.

[40] Rafiei Sardooi E, Azareh A, Choubin B, Barkhori S, Singh VP, Shamshirband S (2019). Applying the remotely sensed data to identify homogeneous regions of watersheds using a pixel-based classification approach. Appl Geogr.

[41] Siabi Z, Falahatkar S, Alavi SJ (2019). Spatial distribution of $${\text{X}}_{{{\text{CO}}_{{2}} }}$$ using OCO-2 data in growing seasons. J Environ Manage.

[42] Falahatkar S, Mousavi SM, Farajzadeh M (2017). Spatial and temporal distribution of carbon dioxide gas using GOSAT data over IRAN. Environ Mon Assess.

[43] Mohammed GH, Colombo R, Middleton EM, Rascher U, van der Tol C, Nedbal L, Goulasf Y, Pérez-Priegog O, Dammh A, Meronij M, Joinerc J, Cogliatib S, Verhoefe W, Malenovskýk Z, Gastellu-Etchegorryl J-P, Millerm JR, Guantern L, Morenoo J, Moyaf I, Berryp JA, Frankenbergq C, Zarco-Tejada PJ (2019). Remote sensing of solar-induced chlorophyll fluorescence (SIF) in vegetation: 50 years of progress. Remote Sensing Environ.

[44] Duveiller G, Filipponi F, Walther S, Köhler P, Frankenberg C, Guanter L, Cescatti A (2020). A spatially downscaled sun-induced fluorescence global product for enhanced monitoring of vegetation productivity. Earth System Sci Data.

[45] Campbell PKE, Huemmrich KF, Middleton EM, Ward LA, Julitta T, Daughtry CST, Burkart A, Russ AL, Kustas WP (2019). Diurnal and seasonal variations in chlorophyll fluorescence associated with photosynthesis at leaf and canopy scales. Remote Sens.

[46] Sun Y, Frankenberg C, Jung M, Joiner J, Guanter L, Köhler P, Magney T (2018). Overview of solar-induced chlorophyll fluorescence (SIF) from the orbiting Carbon observatory-2: retrieval, cross-mission comparison, and global monitoring for GPP. Remote Sens Environ.

[47] Rolim GS, Aparecido LEO (2016). Camargo, Köppen and Thornthwaite climate classification systems in defining climatical regions of the state of São Paulo, Brazil. Int J Climatol.

[48] Taiz L, Zeiger E. Fisiologia Vegetal. 4th ed. Editora Artmed; 2009

[49] Frankenberg C, O’Dell C, Guanter L, McDuffie J (2012). Remote sensing of near-infrared chlorophyll fluorescence from space in scattering atmospheres: implications for its retrieval and interferences with atmospheric CO_2_ retrievals. Atmos Meas Tech.

[50] Ishizawa M, Mabuchi K, Shirai T, Inoue M, Morino I, Uchino O, Yoshida Y, Belikov D, Maksyutov S (2016). Inter-annual variability of summertime CO_2_ exchange in Northern Eurasia inferred from GOSAT $${\text{X}}_{{{\text{CO}}_{{2}} }}$$. Environ Res Lett.

[51] Hansen R, Mander Ü, Soosaar K (2013). Greenhouse gas fluxes in an open air humidity manipulation experiment. Landscape Ecol.

[52] Snyder RL, Spano D, Schwartz M (2013). Phenology and evapotranspiration. Phenology: an integrative environmental science.

[53] Aparecido LEDO, Ferreira RB, Rolim GDS, SouzaDe BS, SouzaDe PS (2017). Nonlinear agrometeorological models for estimating lychee fruit growth. Rev Bras Frutic.

[54] Kovalskyy V, Henebry GM, Roy DP, Adusei B, Hansen M, Senay G, Mocko DM (2013). Evaluation of a coupled event-driven phenology and evapotranspiration model for croplands in the United States northern Great Plains. J Geophysical Res Atmos.

[55] CONAB, Companhia Nacional de Abastecimento. Calendario de plantio e colheita de grãos no Brasil 2019. 2019. https://www.conab.gov.br/institucional/publicacoes/outras-publicacoes/item/7694-calendario-agricola-plantio-e-colheita.

[56] Shekhar A, Chen J, Paetzold JC, Dietrich F, Zhao X, Bhattacharjee S, Ruisinger V, Wofsy SC (2020). Anthropogenic CO_2_ emissions assessment of Nile Delta using $${\text{X}}_{{{\text{CO}}_{{2}} }}$$ and SIF data from OCO-2 satellite. Environ Res Lett.

[57] Guo M, Wang X, Li J, Yi K, Zhong G, Tani H (2012). Assessment of global carbon dioxide concentration using MODIS and GOSAT data. Sensors.

[58] Taylor TE, Eldering A, Merrelli A, Kiel M, Somkuti P, Cheng C, Rosenberg R, Fisher B, Crisp D, Basilio R, Bennett M, Cervantes D, Chang A, Dang L, Frankenberg C, Haemmerle VR, Keller GR, Kurosu T, Laughner JL, Yu S (2020). OCO-3 early mission operations and initial (vEarly) $${\text{X}}_{{{\text{CO}}_{{2}} }}$$ and SIF retrievals. Remote Sens Environ.

[59] Rolim GS, de Oliveira Aparecido LE, de Souza PS (2020). Climate and natural quality of Coffea Arabica L. drink. Theor Appl Climatol.

[60] de Oliveira Aparecido LE, de Souza Rolim G, da Silva Cabral de Moraes, JR.  (2020). Validation of ECMWF climatic data, 1979–2017, and implications for modelling water balance for tropical climates. Int J Climatol.

[61] White JW, Hoogenboom G, Stackhouse PW, Hoell JM (2008). Evaluation of NASA satellite- and assimilation model-derived long-term daily temperature data over the continental US. Agric For Meteorol.

[62] Zhang Y, Joiner J, Hamed Alemohammad S, Zhou S, Gentine P (2018). A global spatially contiguous solar-induced fluorescence (CSIF) dataset using neural networks. Biogeosciences.

[63] Yu L, Wen J, Chang CY, Frankenberg C, Sun Y (2019). High-resolution global contiguous SIF of OCO-2. Geophys Res Lett.

[64] SEADE—Fundação estadual de Análise de dados—Perfil dos municípios paulista. 2020. https://perfil.seade.gov.br/

[65] Camargo FP et al (2020). Previsões e Estimativas das Safras Agrícolas do Estado de São Paulo, Ano Agrícola 2019/20. Análises e Indicadores do Agronegócio, São Paulo, v. 15, n. 9, set. http://www.iea.sp.gov.br/out/TerTexto.php?codTexto=14839

[66] Camargo AP, Heldwein AB, Schneider FM, Buriol GA, Petter Medeiros SL, Estefanel V (1991). Classificação climática para zoneamento de aptidão agroclimática. Congresso Brasileiro de Agrometeorologia.

[67] Nikitenko AA, Timofeev YM, Berezin IA, Poberovskii AV, Virolainen YA, Polyakov AV (2020). The analysis of OCO-2 satellite measurements of CO_2_ in the vicinity of Russian cities. Atmos Ocean Opt.

[68] Massie ST, Cronk H, Merrelli A, O’Dell C, Sebastian Schmidt K, Chen H, Baker D (2021). Analysis of 3D cloud effects in OCO-2 $${\text{X}}_{{{\text{CO}}_{{2}} }}$$ retrievals. Atmos Meas Tech.

[69] Mandrake L, O’Dell C, Wunch D, Wennberg PO, Fisher B, Osterman GB, Eldering A. (2015). Lite files, warn level and bias correction determination. Tech. Rep., Jet Propul. Lab., California Inst. of Technol., Pasadena, Calif.

[70] AppEEARS Team. 2020. Application for extracting and exploring analysis ready samples (AppEEARS). Ver. 6. NASA EOSDIS Land processes distributed active archive Center (LP DAAC), USGS/Earth resources observation and science (EROS) center, Sioux Falls, South Dakota, USA. https://lpdaacsvc.cr.usgs.gov/appeears/. Accessed Nov 11 2020.

[71] Stackhouse PW Jr, Westberg D, Chandler WS, Zhang T, Hoell JM (2015). Prediction of worldwide energy resource (POWER) agroclimatology methodology, version 1.1.0, May 30. NASA Langley Research Center.

[72] Gujarati DN, Porter DC. 2011. Econometria básica-5. Amgh Editora.

[73] R Core team 2021. R: a language and environment for statistical computing. R Foundation for Statistical Computing, Vienna, Austria. www.R-project.org/.

[74] Gareth J, Daniela W, Trevor H, Robert T (2013). An introduction to statistical learning: with applications in R.

